# Prolapsing left atrial mass causing severe pulmonary hypertension in a new cardiac service in a rural hospital in Cameroon: a case report

**DOI:** 10.11604/pamj.2022.41.196.12231

**Published:** 2022-03-11

**Authors:** Clovis Nkoke, Christelle Makoge

**Affiliations:** 1 Buea Regional Hospital, Buea, Cameroon,; 2 Faculty of Medicine and Biomedical Sciences, University of Yaoundé I, Yaoundé, Cameroon

**Keywords:** Left atrial mass, myxoma, pulmonary hypertension, rural, case report

## Abstract

We report a case of a left atrial mass in a 62-year-old patient with no relevant past history. He presented with dyspnea of 1 year duration. Clinical examination revealed a blood pressure of 130/82mmHg, a heart rate of 80 beats per minute. The heart sounds S1 and S2 were normal with no added sounds. Electrocardiogram showed a normal sinus rhythm at 78 beats per minute with premature ventricular contractions. Two dimensional echocardiography revealed a large mobile mass attached to the interatrial septum occupying the most of the left atrium and prolapsing into the left ventricle during diastole. There was dilatation of the right atrium and right ventricle with elevated pulmonary artery systolic pressure (85mmHg). The mean transmitral pressure gradient was 5.5mmHg. The mass was compatible with a myxoma. The patient was sent for surgical resection of the mass but this could not be performed due to financial constraints.

## Introduction

Atrial myxomas are the most frequent cardiac tumors. About 75% of myxomas are found in the left atrium, attached to the interatrial septum. The clinical presentation may be non-specific. Depending on the size and location; it may cause mitral valve obstruction and pulmonary hypertension [1]. We report a case of an unusually large left atrial mass presumed to be a myxoma in a 62-year-old man that caused pulmonary hypertension and mitral valve obstruction.

## Patient and observation

**Patient information:** a 62-year-old man with no relevant past history presented with a one year history of increasing dyspnea on exertion. There was no chest pain on exertion, no palpitations, no lower limbs oedema. He had no significant pas medical history and was not on any regular medications.

**Clinical findings:** on physical examination, the patient was in no apparent distress. His blood pressure was 130/82 mmHg and his heart rate was 80 beats per minute. The heart sounds S1 and S2 were normal, there were no added heart sounds. The lungs were clear. There was no lower limbs oedema. The rest of the physical examination was unremarkable.

**Diagnostic assessment:** a 12 lead electrocardiogram (ECG) showed a normal sinus rhythm at 78 beats per minute with premature ventricular contractions. There were no signs of myocardial ischemia. A transthoracic echocardiographic examination showed the presence of an echogenic, highly mobile mass stemming from the interatrial septum . The mass 23mm x 56mm in size, was prolapsing toward the left ventricle and producing a transmitral gradient of 5.5mmHg. The right ventricle and right atrium were mildly dilated. There was moderate tricuspid regurgitation with an estimated pulmonary artery systolic pressure of 85mmHg. The left ventricle was normal. The mass presumed to a myxoma.

**Therapeutic intervention:** the patient was sent to the cardiac surgical center for surgical resection and confirmation of the diagnosis but this could not be performed due to financial constraints. This was the only beneficial therapeutic intervention that could be proposed to the patient at that time.

**Informed consent:** the patient was informed about the case report, why his case was peculiar and the authors' interest in publishing his case. He willingly gave informed consent to allow the authors to use echo images for this case report.

**Patient´s consent:** informed consent was obtained from the patient for us to use the echo images.

**Ethical approval:** consent was obtained from the patient´s guardian for the publication of the case.

## Discussion

We have reported the case of a left atrial mass compatible with a left atrial myxoma. The symptoms of left atrial myxoma are usually non-specific making early diagnosis difficult [2]. In a series of 149 cases, Meng *et al*. reported a 4% rate of asysmptomatic left atrial myxomas [3]. As seen in this case report, atrial myxomas may develop into giant masses without producing any symptoms. Symptoms may develop late in the course of tumour development and may be vague or nonspecific. The main presenting symptom in our patient was dyspnea. In patients with left atrial myxomas, symptoms of left-sided heart failure, such as dyspnea on exertion, may progress to orthopnea, paroxysmal nocturnal dyspnea or pulmonary edema [4, 5]. Echocardiography is useful for detecting cardiac causes for dyspnea and is readily available in all settings. As the early surgical death rate is low and long-term results are good, cardiac myxoma should be removed as soon as a diagnosis is made. Our patient was sent for surgical resection but due to financial limitations it was not performed.

## Conclusion

The diagnosis of left atrial myxomas may be easily missed, but echocardiography is a simple way of establishing the diagnosis. Excision of the tumor usually results in marked improvement of symptoms. But treatment can be challenging in resource limited settings.
